# Supplementary material for: A method for site-specifically tethering the enzyme urease to DNA origami with sustained activity
Source: PLoS One. 2025 Apr 21;20(4):e0319790. doi: 10.1371/journal.pone.0319790 (PMC12011258; doi:10.1371/journal.pone.0319790)
Supplement: S1 File — Supporting information text provides detailed protocols for DNA-origami design, folding, purification, and imaging. (PDF) [file pone.0319790.s001.pdf]

Supporting information

DNA origami techniques

Designing DNA origami The full design for the six-helix bundle is available on Nanobase as structure 249 [1].

Folding DNA origami Each DNA origami particle is folded by mixing 50 nM of p8064 scaffold DNA, which has 8064 nucleotides (Tilibit), and 200 nM each of staple strands with folding buffer and annealed through a temperature ramp starting at 65 °C for 15 minutes, then 54 to 51 °C, −1 °C per hour. Our folding buffer contains 5 mM Tris Base, 1 mM EDTA, 5 mM NaCl, and 5 mM $MgCl_2$. We use a Tetrad (Bio-Rad) thermocycler for annealing the solutions.

Agarose gel electrophoresis To assess the outcome of folding, we perform agarose gel electrophoresis. Gel electrophoresis requires the preparation of the gel and the buffer. The gel is prepared by heating a solution of 1.5% wt/wt agarose, 0.5x TBE to boiling in a microwave. The solution is cooled to 60 °C. At this point, we add $MgCl_2$ solution and SYBR-safe (Invitrogen) to adjust the concentration of the gel to 5.5 mM $MgCl_2$ and 0.5x SYBR-safe. The solution is then quickly cast into an Owl B2 gel cast, and further cooled to room temperature. The buffer solution contains 0.5x TBE and 5.5 mM $MgCl_2$. Agarose gel electrophoresis is performed at 90 V for 1.5 hours at 4 °C. The gel is then scanned with a Typhoon FLA 9500 laser scanner.

Gel purification and resuspension After folding, DNA-origami particles are purified to remove all excess staples and misfolded aggregates using gel purification. The DNA origami are run through an agarose gel (now at a 1xSYBR-safe concentration for visualization) prepared using a custom gel comb, which can hold around 4 mL of solution per gel. We use a black light box (Hall Productions BL1012) to identify the gel band containing the folded DNA origami. The folded origami band is then extracted using a razor blade and cut into pieces. We place the gel pieces into a Freeze 'N Squeeze spin column (Bio-Rad), freeze it in a –80 °C freezer for 30 minutes, thaw at room temperature, and then spin the solution down for 5 minutes at 13,000 x*g*.

Next, we concentrate the solution through ultrafiltration [2]. First, a 0.5-mL Amicon 100 kDA ultrafiltration spin column is equilibrated by centrifuging down 0.5 mL of the folding buffer at 5,000 x*g* for 7 minutes. Then, the DNA-origami solution is added up to 0.5 mL and centrifuged at 14,000 x*g* for 15 minutes. Finally, we flip the filter upside down into a new Amicon tube and spin down the solution at 1,000 x*g* for 2 minutes. The concentration of the DNA origami is measured using a Nanodrop (Thermofisher), assuming that the solution consists only of well-folded particles that are each 8064 base pairs.

Negative stain TEM We first prepare a solution of uranyl formate (UFo). Millipore water is boiled to deoxygenate it and then mixed with uranyl formate powder to create a 2% wt/wt UFo solution. The solution is covered with aluminum foil to avoid light exposure, then vortexed vigorously for 20 minutes. The solution is filtered using a 0.2 µm filter. The solution is divided into 0.2-mL aliquots, which are stored in a –80 °C freezer until further use.

Prior to each negative-stain TEM experiment, a 0.2-mL aliquot of UFo is taken out from the freezer to thaw at room temperature. We add 4 µL of 1 M NaOH to precipitate the UFo and vortex the solution vigorously for 15 seconds. The solution is

centrifuged at 4 °C and 16,000 xg for 8 minutes. We extract 170 μL of the supernatant for staining and discard the rest.

The EM samples are prepared using FCF400-Cu grids. We glow discharge the grid prior to use at −20 mA for 30 seconds at 0.1 mbar, using a Quorum Emitech K100X glow discharger. We place 4 μL of the sample on the grid for 1 minute to allow adsorption of the sample to the grid. During this time 5 μL and 18 μL droplets of UFo solution are placed on a piece of parafilm. After the adsorption period, the remaining sample solution is blotted on a Whatman filter paper. We then touch the carbon side of the grid to the 5 μL drop and blot it away immediately to wash away any buffer solution from the grid. This step is followed by picking up the 18 μL UFo drop onto the carbon side of the grid and letting it rest for 30 seconds to deposit the stain. The UFo solution is then blotted to remove excess fluid. Grids are dried for a minimum of 15 minutes before insertion into the TEM.

We image the grids using an FEI Morgagni TEM operated at 80 kV with a Nanosprint5 CMOS camera. The microscope is operated at 80 kV and images are acquired between x8,000 to x28,000.

References

1. Poppleton E, Mallya A, Dey S, Joseph J, Šulc P. Nanobase.org: a repository for DNA and RNA nanostructures. Nucleic Acids Research. 2022;50(D1):D246–D252.

2. Wagenbauer KF, Engelhardt FA, Stahl E, Hechtl VK, Stömmer P, Seebacher F, et al. How we make DNA origami. ChemBioChem. 2017;18(19):1873–1885.
